# Human gut microbiome impacts skeletal muscle mass via gut microbial synthesis of the short‐chain fatty acid butyrate among healthy menopausal women

**DOI:** 10.1002/jcsm.12788

**Published:** 2021-09-01

**Authors:** Wan‐Qiang Lv, Xu Lin, Hui Shen, Hui‐Min Liu, Xiang Qiu, Bo‐Yang Li, Wen‐Di Shen, Chang‐Li Ge, Feng‐Ye Lv, Jie Shen, Hong‐Mei Xiao, Hong‐Wen Deng

**Affiliations:** ^1^ Center for System Biology, Data Sciences, and Reproductive Health, School of Basic Medical Science Central South University Changsha Hunan China; ^2^ Department of Endocrinology and Metabolism The Third Affiliated Hospital of Southern Medical University Guangzhou China; ^3^ Tulane Center of Biomedical Informatics and Genomics, Deming Department of Medicine Tulane University School of Medicine New Orleans LA USA; ^4^ LC‐Bio Technologies Co., Ltd. Hangzhou China; ^5^ Shunde Hospital of Southern Medical University (The First People's Hospital of Shunde) Foshan Guangdong China

**Keywords:** Gut microbiome, Host genetics, Gut microbial synthesis of the SCFA butyrate, Skeletal muscle mass

## Abstract

**Background:**

Increasing evidence suggests that human gut microbiome plays an important role in variation of skeletal muscle mass (SMM). However, specific causal mechanistic relationship of human gut microbiome with SMM remains largely unresolved. Understanding the causal mechanistic relationship may provide a basis for novel interventions for loss of SMM. This study investigated whether human gut microbiome has a causal effect on SMM among Chinese community‐dwelling healthy menopausal women.

**Methods:**

Estimated SMM was derived from whole‐body dual‐energy X‐ray absorptiometry. We performed integrated analyses on whole‐genome sequencing, shotgun metagenomic sequencing, and serum short‐chain fatty acids (SCFAs), as well as available host SMM measurements among community‐dwelling healthy menopausal women (*N* = 482). We combined the results with summary statistics from genome‐wide association analyses for human gut microbiome (*N* = 952) and SMM traits (*N* = 28 330). As a prerequisite for causality, we used a computational protocol that was proposed to measure correlations among gut metagenome, metabolome, and the host trait to investigate the relationship between human gut microbiome and SMM. Causal inference methods were applied to assess the potential causal effects of gut microbial features on SMM, through one‐sample and two‐sample Mendelian randomization (MR) analyses, respectively.

**Results:**

In metagenomic association analyses, the increased capacity for gut microbial synthesis of the SCFA butyrate was significantly associated with serum butyrate levels [Spearman correlation coefficient (SCC) = 0.13, *P* = 0.02] and skeletal muscle index (SCC = 0.084, *P* = 0.002). Of interest was the finding that two main butyrate‐producing bacterial species were both positively associated with the increased capacity for gut microbial synthesis of butyrate [ *Faecalibacterium prausnitzii* (SCC = 0.25, *P* = 6.6 × 10^−7^) and *Butyricimonas virosa* (SCC = 0.15, *P* = 0.001)] and for skeletal muscle index [ *F. prausnitzii* (SCC = 0.16, *P* = 6.2 × 10^−4^) and *B. virosa* (SCC = 0.17, *P* = 2.4 × 10^−4^)]. One‐sample MR results showed a causal effect between gut microbial synthesis of the SCFA butyrate and appendicular lean mass (*β* = 0.04, 95% confidence interval 0.029 to 0.051, *P* = 0.003). Two‐sample MR results further confirmed the causal effect between gut microbial synthesis of the SCFA butyrate and appendicular lean mass (*β* = 0.06, 95% confidence interval 0 to 0.13, *P* = 0.06).

**Conclusions:**

Our results may help the future development of novel intervention approaches for preventing or alleviating loss of SMM.

## Introduction

Sarcopenia, characterized by loss of skeletal muscle mass (SMM) and function that occurs with ageing, is an age‐related skeletal muscle disorder, leading to substantially increased risk of falls, fractures, and disability.^1^, ^2^ Although some studies have shown that the combined resistance training/protein/vitamin D/calcium intervention was feasible and effective for tackling sarcopenia,^3^, ^4^ the aetiology of sarcopenia is poorly understood to date, and more efficient treatments and drugs are still much needed for sarcopenia. With ageing, it is important to understand the pathophysiological mechanisms and improve strategies for early identification and intervention of sarcopenia.

Increasing evidence suggests that human gut microbiome (HGM) plays a key role in SMM and function.^5^, ^6^ For example, Lahiri *et al*.^5^ found that germ‐free mice (lacking gut microbiota) displayed reduced SMM and decreased expression of genes associated with skeletal muscle growth and function, compared with pathogen‐free mice (had gut microbiota). Moreover, transplanting the gut microbiota of pathogen‐free mice into the germ‐free mice restored muscle mass.^5^ An intervention study showed that supplementation with prebiotics (Darmocare Pre^®^, a mixture of inulin plus fructooligosaccharides) could reduce the level of fatigue and improve the level of muscle strength in 60 old individuals (aged 65 and over).^6^ However, the significance of HGM on SMM and function has not been directly investigated in humans. In addition, as HGM is considered to be dynamic and can be influenced by host conditions,^7^ it is essential to discriminate gut microbial alterations that are causal for disease from those that are a consequence of disease or its treatment and from those that show a statistical correlation due to confounding or pleiotropy.

Mendelian randomization (MR) offers an opportunity to distinguish between causal and non‐causal effects from cross‐sectional studies.^8^ Recently, MR approach has been applied to investigate the relationship of gut microbiome on cardiovascular diseases.^9^, ^10^ Mounting studies have demonstrated that it is possible to detect variants in the host genome that influence HGM variation.^10^, ^11^ These findings allowed us to deploy MR approach to infer the causal relationship by investigating whether genetic predictors of HGM would influence SMM.

**Figure 1:**
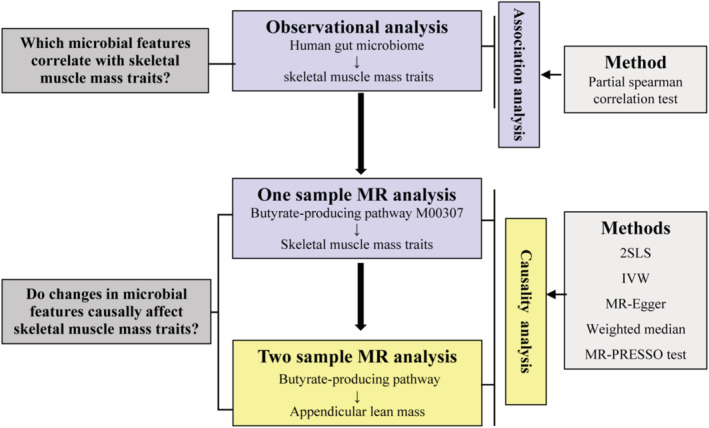
Study design overview. The light blue was analysed in the individual‐level data (*N* = 482). The light yellow was using the publicly available data, carried out with up to nine genetic predictors and their effect sizes from the LifeLines‐DEEP cohort (952 samples) and the GEnetic Factors for OSteoporosis (GEFOS) summary statistics (appendicular lean mass sample size = 28 330). 2SLS, two‐stage least squares regression; IVW, inverse variance‐weighted method; MR, Mendelian randomization; MR‐PRESSO, MR pleiotropy residual sum and outlier test. M00307 refers to a microbial pathway involved in gut microbial synthesis of short‐chain fatty acid butyrate (M00307: pyruvate oxidation, pyruvate → acetyl‐CoA).

In the present study, we aimed to investigate whether HGM (bacterial species or gut metabolic pathways) has a causal effect on SMM traits. An overview of the study design was illustrated in *Figure* 1. First, we performed a cross‐sectional study to detect the association between gut microbial features (gut metagenomics species or gut metabolic pathways) and SMM traits by leveraging information from 482 Chinese menopausal women for whom shotgun metagenomic sequencing, serum short‐chain fatty acid (SCFA) levels, and host SMM were performed/measured. Benefited from whole‐genome sequencing of the individuals in this study, we conducted one‐sample MR analysis to explore the causal association between gut microbial synthesis of SCFA butyrate and SMM traits. Then, we conducted two‐sample MR analysis to further partially confirm the findings of the cross‐sectional study by using publicly available summary statistics of genome‐wide association analysis for HGM and SMM.

## Materials and methods

### Individual‐level data

This study was approved by the Third Affiliated Hospital of Southern Medical University at Guangzhou (201711012) on 27 November 2017. All participants provided written informed consent at enrolment. The inclusion criteria included (i) aged 40 years or older, (ii) being in menopause stage (experiencing an absence of menstruation or amenorrhea for at least 3 months; self‐reported), and (iii) had lived in Guangzhou City for at least 3 months. Briefly, exclusion criteria included the use of antibiotics, oestrogens, or anticonvulsant medications, which may affect HGM in the past 3 months, as well as other diseases that could lead to secondary osteoporosis. In addition, individuals with missing data were also excluded in the current study. Detailed information on the study design has been described previously.^12^ The individuals of this study were recruited for shotgun metagenomic sequencing of gut microbiome and whole‐genome sequencing of human hosts. In addition, measurement of SCFAs in fasting serum was carried out through gas chromatography–mass spectrometry. The details of individual‐level data were described in the Supporting Information. The quality control process for each individual‐level data was described in the respective sections of the Supporting Information. We used whole‐body dual‐energy X‐ray absorptiometry (Lunar, GE Healthcare, Chicago, Illinois, USA) to extract SMM traits, including appendicular lean mass (ALM) and skeletal muscle index (SMI, calculated as ALM in kilograms divided by body mass index).

After excluding any individuals with missing data on one of the multi‐omics data, we finally obtained multi‐omics data from 482 Chinese Han menopausal women for the subsequent analysis, including human whole‐genome sequencing, gut microbiome shotgun metagenomic sequencing, and human serum SCFAs levels, as well as host SMM traits. Age, gender, and dietary and lifestyle factors were self‐reported. Characteristics of individuals in this study were described in *Table* 1.

**Table 1:** Characteristics of individuals in individual‐level data

Variable	Participant no. (%) (*n* = 482)	Coding^a^
Female (%)	482 (100)	0
Age, median (min–max), years	53 (41–65)	
Height, median (min–max), cm	158.0 (142.0–173.0)	
BMI, median (min–max), kg/m^2^	22.56 (16.42–33.73)	
ALM, median (min–max), kg	14.69 (10.22–20.69)	
SMI, median (min–max), m^2^	0.64 (0.43–1.00)	
Alcohol status, no. (%)		
Never	431 (89.4)	1
Sometimes	51 (10.6)	2
Second‐hand smoke status, no. (%)		
Rarely	314 (65.1)	1
<1 h/day	89 (18.5)	2
≥1 and <2 h/day	36 (7.5)	3
≥2 and <4 h/day	35 (7.3)	4
≥4 h/day	8 (1.7)	5
Milk drink status, no. (%)		
0 mL/day	317 (65.8)	1
>250 mL/day	156 (32.4)	2
≥250 and <500 mL/day	6 (1.2)	3
≥500 mL/day	3 (0.6)	4
Soybean milk drink status, no. (%)		
0 mL/day	436 (90.5)	1
<250 mL/day	43 (8.9)	2
≥250 and <500 mL/day	2 (0.4)	3
≥500 mL/day	1 (0.2)	4
Coffee drink status, no. (%)		
0 mL/day	459 (95.2)	1
<250 mL/day	21 (4.4)	2
≥250 and <500 mL/day	1 (0.2)	3
≥500 mL/day	1 (0.2)	4
Tea drink status, no. (%)		
0 mL/day	308 (63.9)	1
<250 mL/day	61 (12.7)	2
≥250 and <500 mL/day	49 (10.2)	3
≥500 and <750 mL/day	28 (5.8)	4
≥750 mL/day	36 (7.5)	5
Exercise status, no. (%)		
False	142 (29.5)	1
True	340 (70.5)	2
Education level, no. (%)		
Middle school	137 (28.4)	1
High school	144 (29.9)	2
Bachelor's degree	87 (18.0)	3
College	101 (21.0)	4
Master's degree	10 (2.1)	5
Doctoral degree	3 (0.6)	6
Red meat intake, no. (%)		
<50 g/day	190 (39.4)	1
50–100 g/day	255 (52.9)	2
100–200 g/day	33 (6.8)	3
≥200 g/day	4 (0.8)	4
Stress status, no. (%)		
Never	253 (52.5)	1
Sometimes	174 (36.1)	2
Often	55 (11.4)	3
Anxiety, no. (%)		
Never	231 (47.9)	1
Sometimes	203 (42.1)	2
Often	48 (10.0)	3
Fatigue, no. (%)		
Never	286 (59.3)	1
Sometimes	159 (33.0)	2
Often	37 (7.7)	3
Nerves, no. (%)		
Never	288 (59.8)	1
Sometimes	168 (34.9)	2
Often	26 (5.4)	3
Estradiol level, no. (%)		
<18.35 pmol/L	322 (66.8)	1
18.35–91.75 pmol/L	114 (23.6)	2
≥91.75 pmol/L	46 (9.5)	3

ALM, appendicular lean mass; BMI, body mass index; SMI, skeletal muscle index.

^a^ To facilitate the description of categorical traits and analyse the current study, we revalued the categorical traits using the coding method. For example, second‐hand smoke status is a categorical and ordinal variable, thus categorized as an ordinal variable (1, 2, 3, 4, and 5).

### Summary statistic data

Summary statistics data for two‐sample MR analysis were obtained from published genome‐wide association studies (GWASs) for HGM (*N* = 952) and ALM (*N* = 28 330), both from independent large cohorts of European‐descent individuals.^9^, ^13^ The characteristics of summary statistic data were presented in *Table* S1.

### Metagenomic association analysis

In metagenomic association analysis, we performed a cross‐sectional study to detect the association between gut microbial features (gut metagenomics species or gut metabolic pathways) and SMM traits. We used a computational protocol that was proposed to measure correlations among gut metagenome, metabolome, and the host trait.^14^ In brief, we used Spearman correlation test to investigate the correlation of metagenomic species with SMM traits, given the skewed distribution of metagenomic data. Considering the effects of diet and lifestyle factors on gut microbial features, we added age and 14 diet and lifestyle factors as covariates for association analysis. Mann–Whitney *U* test was used to test the associations of microbial functional pathways with SMM traits, where the ranks of KOs within a given KEGG functional pathway were compared with the ranks of all other KOs.^14^ Given the characteristics of sparsity that is presented in gut microbial features across samples, we only included metagenomic species and functional pathways present in at least five individuals to avoid artefactual results in our study. The results were considered significant when the false discovery rate (FDR) *P* was <0.1.

### Genome‐wide association analysis of gut microbial features

We tested the associations between host genetics and gut microbial features using linear or logistic model based on the relative abundance of gut microbial features. The relative abundance of gut microbial features with occurrence rate over 95% of subjects in the study was transformed by the natural logarithm, so that the abundance of gut microbial features could be treated as a quantitative trait (AB model). Otherwise, we dichotomized gut microbial features into presence/absence (P/A) patterns to prevent zero inflation,^10^ and then the relative abundance of features could be treated as a dichotomous trait (P/A model). Next, for the common SNP variants (MAF ≥ 1%), we performed a standard single variant‐based GWAS analysis via PLINK (Version 1.9) using a linear model for quantitative features or a logistic model for dichotomous features. Given the effects of diet and lifestyle factors on gut microbial features, we included age and 14 diet and lifestyles, as well as the Top 4 principal components (PCs) of population structure as covariates for GWAS analysis.

### One‐sample Mendelian randomization analysis

For each of the whole genome‐wide association results of gut microbial features, we selected genetic variants that showed association at *P* < 1 × 10^−5^ and performed linkage disequilibrium (LD) estimation with a threshold of LD *r* ^2^ < 0.1 for clumping analysis to obtain independent genetic predictors, consistent with previous studies.^9^, ^15^ Then, an unweighted polygenic risk score (PRS) was calculated for each individual using independent genetic variants from the whole genome‐wide association results. Each variant was recoded as 0, 1, and 2, depending on the number of trait‐specific risk increasing alleles carried by an individual. We performed instrumental variables analyses employing two‐stage least squares regression method. Two‐stage least squares regression was performed using ‘ivreg’ command from the AER package in R (R Foundation for Statistical Computing, Vienna, Austria). In brief, linear regression was performed with SMM traits and genetically predicted gut microbial features (i.e. unweighted PRS for gut microbial features based on the instruments), adjusting for age, 14 diet and lifestyle factors, and Top 4 PCs of population structure.

### Two‐sample Mendelian randomization analysis

Consistent with one‐sample MR analysis, the same threshold (LD *r* ^2^ < 0.1 and *P* < 1 × 10^−5^) was used to include more genetic variants and maximize the strength of instruments. We used the TwoSampleMR package to facilitate MR analyses.^16^ We used the inverse variance‐weighted (IVW) meta‐analyses as the primary method, which uses weighted linear regression and is equivalent to two‐stage least squares or allele score analysis using individual‐level data.^17^ It provides a statistically consistent estimator of the true causal effect as long as all genetic variants are valid instrumental variables.

Sensitivity analyses for the MR were performed using the weighted median and the MR–Egger method.^18^, ^19^ The weighted median method allows some genetic variants to be invalid instruments and is robust to outliers in the genetic variant‐specific causal effect estimates. The MR–Egger method allows all genetic variants to be invalid but requires that the pleiotropic effects related to outcome risk through pleiotropic pathways are uncorrelated with the genetic variant‐exposure associations and is also sensitive to outliers. The intercept test of the MR–Egger method was used to test for horizontal pleiotropy. Additionally, we assessed the presence of horizontal pleiotropy by using the MR‐PRESSO Global test.^20^ Cochran *Q* test was adopted to quantify the between‐SNP heterogeneity in the IVW MR analysis, with *P* < 0.05 deemed as statistically significant heterogeneous.

## Results

### Gut microbial features associated with skeletal muscle mass traits

Leveraging the information from the 482 Chinese menopausal women, we assessed the associations of gut microbial features (gut metagenomics species or gut metabolic pathways) with host SMM traits, focusing the results on gut microbial features that were correlated (FDR *P* < 0.1) with at least one of SMM traits (i.e. ALM or SMI).

In the association analysis of gut metabolic pathways, 19 of the 646 gut metabolic pathways (3%) were associated (FDR *P* < 0.1) with one or more of SMM traits after adjusting for age and 14 diet and lifestyle factors (*Table* 2). Among these results, we observed a specific pathway (M00307: pyruvate oxidation, pyruvate → acetyl‐CoA), which is involved in anaerobic fermentation processes and gut microbial synthesis of the SCFAs (butyrate and acetate).^21^ The study had shown that the bacterial butyrate synthesis pathways are prevalent and important for a healthy gut^22^; thus, we focused the effect of this bacterial butyrate synthesis pathway on SMM traits in this study. We found that the M00307 was associated with increased SMI [Spearman correlation coefficient (SCC) = 0.08, *P* = 0.002, FDR *P* = 0.08]. Previous studies have shown that the major end products of M00307 fermentation pathways are butyrate and acetate.^21^, ^23^ Thus, we directly correlated the relative abundance of M00307 to the levels of host serum butyrate and acetate. The relative abundance of M00307 was significantly correlated with serum butyrate levels (SCC = 0.13, *P* = 0.02) and acetate (SCC = 0.15, *P* = 0.03). However, the association of serum butyrate levels on ALM (SCC = 0.01, *P* = 0.81) and SMI (SCC = 0.06, *P* = 0.05) did not reach statistical significance. We hypothesize that the effect of butyrate on regulating SMM may depend more on physiological concentrations of butyrate in muscle tissue rather than in serum. Skeletal muscle tissues are known to possess cell surface receptors for SCFAs and exhibit beneficial alterations phenotype and physiology.^24^, ^25^

**Table 2:** Associations between gut microbial modules and skeletal muscle mass traits in 482 menopausal Chinese individuals

Module ID	Module description	ALM			SMI		
		SCC	*P*	FDR	SCC	*P*	FDR
M00008	Entner–Doudoroff pathway, glucose‐6P → glyceraldehyde‐3P + pyruvate	−0.067	3.33E − 03	0.187	−0.071	2.20E − 03	0.083
M00061	d‐Glucuronate degradation	−0.040	2.11E − 02	0.274	−0.080	4.85E − 04	0.074
M00063	CMP–KDO biosynthesis	−0.030	1.11E − 01	0.487	−0.077	4.04E − 03	0.097
M00090	Phosphatidylcholine (PC) biosynthesis, choline → PC	0.048	3.34E − 03	0.187	0.074	3.93E − 03	0.097
M00113	Jasmonic acid biosynthesis	−0.027	1.51E − 02	0.256	−0.043	3.08E − 03	0.083
M00178	Ribosome, bacteria	0.046	3.00E − 13	0.000	0.043	7.72E − 08	0.000
M00179	Ribosome, archaea	0.018	1.20E − 02	0.256	0.017	1.78E − 03	0.083
M00275	PTS system, cellobiose‐specific II component	−0.091	5.25E − 03	0.193	−0.126	3.08E − 03	0.083
M00276	PTS system, mannose‐specific II component	−0.058	6.66E − 03	0.193	−0.092	1.66E − 03	0.083
M00277	PTS system, *N*‐acetylgalactosamine‐specific II component	−0.039	3.09E − 02	0.327	−0.126	7.20E − 04	0.077
M00279	PTS system, galactitol‐specific II component	−0.088	7.19E − 03	0.193	−0.142	2.92E − 03	0.083
M00283	PTS system, ascorbate‐specific II component	−0.082	6.58E − 03	0.193	−0.135	3.03E − 03	0.083
M00307	Pyruvate oxidation, pyruvate → acetyl‐CoA	0.046	5.10E − 02	0.387	0.084	2.22E − 03	0.083
M00357	Methanogenesis, acetate → methane	0.018	6.83E − 02	0.425	0.057	2.51E − 05	0.006
M00530	Dissimilatory nitrate reduction, nitrate → ammonia	−0.039	4.72E − 03	0.193	−0.050	2.86E − 03	0.083
M00608	2‐Oxocarboxylic acid chain extension, 2‐oxoglutarate → 2‐oxoadipate → 2‐oxopimelate → 2‐oxosuberate	0.046	1.57E − 02	0.256	0.097	1.64E − 03	0.083
M00610	PTS system, d‐glucosaminate‐specific II component	−0.100	9.86E − 04	0.136	−0.124	8.46E − 04	0.077
M00647	Multidrug resistance, efflux pump AcrAB‐TolC/SmeDEF	−0.085	8.43E − 04	0.136	−0.110	1.13E − 03	0.083
M00737	Bacitracin resistance, VraDE transporter	0.014	2.71E − 01	0.634	0.063	2.27E − 03	0.083

ALM, appendicular lean mass; FDR, false discovery rate; PC, principal component; PTS, phosphotransferase; SCC, Spearman correlation coefficient; SMI, skeletal muscle index.

In the association analysis of gut metagenomic species, we found that five of the 612 metagenomic species (0.8%) were associated (FDR *P* < 0.1) with at least one of SMM traits after adjusting for age and 14 diet and lifestyle factors (*Table* 3). Of interest was the finding that two of the five associated metagenomic species are the well‐known butyrate‐producing bacteria, including *Faecalibacterium prausnitzii* of the family Ruminococcaceae and *Butyricimonas virosa* of the family Porphyromonadaceae.^26^, ^27^ Moreover, these two butyrate‐producing bacteria were both correlated with the relative abundance of M00307 pathway, including *F. prausnitzii* (SCC = 0.25, *P* < 6.6 × 10^−7^) and *B. virosa* (SCC = 0.15, *P* = 0.001). In addition, *unclassified Erysipelotrichaceae* (species level) of the family Erysipelotrichaceae showed negatively correlation with both ALM and SMI after adjustment for age and 14 diet and lifestyle factors (*Table* 2).

**Table 3:** Associations between microbial species and skeletal muscle mass traits in 482 menopausal Chinese individuals

MGS	Species	Family	ALM			SMI		
			SCC	*P*	FDR	SCC	*P*	FDR
MGS0059	—	Akkermansiaceae	0.122	8.30E − 03	0.339	0.161	4.67E − 04	0.093
MGS0106	Erysipelotrichaceae unclassified	Erysipelotrichaceae	−0.215	2.98E − 06	0.002	−0.176	1.33E − 04	0.073
MGS0133	*Clostridium* sp. CAG:62	Clostridiaceae	0.081	7.98E − 02	0.539	0.155	7.64E − 04	0.093
MGS0477	*Faecalibacterium prausnitzii*	Ruminococcaceae	0.104	2.49E − 02	0.414	0.158	6.20E − 04	0.093
MGS0595	*Butyricimonas virosa*	Porphyromonadaceae	0.154	8.68E − 04	0.208	0.169	2.37E − 04	0.073

ALM, appendicular lean mass; FDR, false discovery rate; MGS, metagenomics species; SCC, Spearman correlation coefficient; SMI, skeletal muscle index.

From the results of the metagenomics association analysis, there was an implication that, in the study sample of Chinese menopausal women, gut microbiome may have clinical benefit to SMM attributable to gut microbial synthesis of SCFA butyrate.

### Human gut microbiome associated with human genome

We conducted microbiome GWAS (mGWAS) for gut microbial features involving gut microbial synthesis of SCFA butyrate (the M00307 pathway), which was identified to be associated with SMI in the metagenomics association analysis. By applying a conservative inclusion threshold of samples and variants, 482 individuals with 7.39 million common variants (MAF ≥ 1%) were used for mGWAS. We identified one independent variant, rs151213951 (*P* = 2.3 × 10^−8^), reaching genome‐wide significance level (*P* < 5 × 10^−8^) for association with the relative abundance of M00307 pathway (*Table* S2). Additional 26 variants showed suggestive associations (*P* < 1 × 10^−5^) with the relative abundance of M00307 pathway (*Table* S2).

### Effects of gut microbial synthesis of short‐chain fatty acid butyrate on skeletal muscle mass traits

To reveal the potential causality between gut microbial synthesis of SCFA butyrate (the M00307 pathway) and SMM traits, we conducted one‐sample and two‐sample MR analyses, respectively.

For the one‐sample MR analysis, we first identified 27 independent SNPs (LD *r* ^2^ < 0.1 and *P* < 1 × 10^–5^) as instrument variants (*Table* S2) for the relative abundance of the M00307 pathway and constructed the M00307‐PRS by leveraging the 27 independent SNP instruments. We then conducted linear regression analyses for the M00307‐PRS and detected significant association of the M00307‐PRS with ALM [*β* = 0.040, 95% confidence interval (CI) 0.029 to 0.051, *P* = 0.003], but not with SMI (*β* = −0.04, 95% CI −0.11 to 0.029, *P* = 0.24). These results were largely consistent with our findings from the metagenomics association analysis where the M00307 pathway was significantly associated with SMI (SCC = 0.08, *P* = 0.002) and ALM (SCC = 0.046, *P* = 0.05), as ALM and SMI were closely related though not identical traits with both having the common measures of SMM.^28^, ^29^

**Figure 2:**
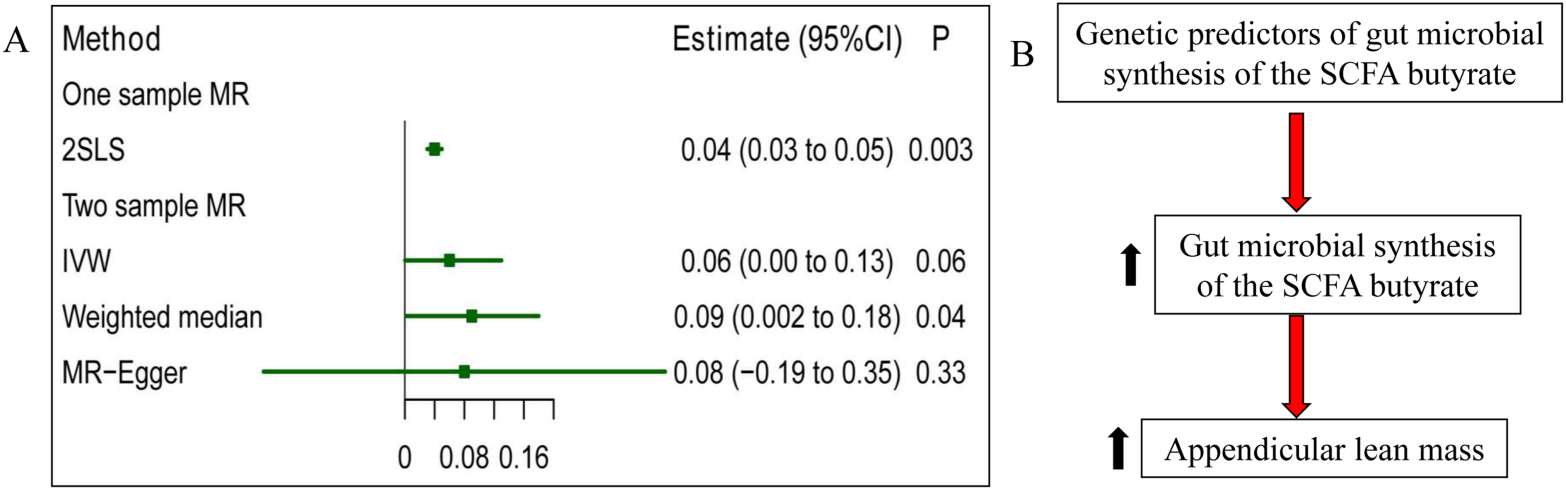
Causal effect of gut microbial synthesis of the short‐chain fatty acid (SCFA) butyrate on appendicular lean mass (ALM). (*A*) Forest plot represented the effect of per 1‐SD increase in gut microbial synthesis of the SCFA butyrate abundance on ALM, as estimated using one‐sample Mendelian randomization (MR) analysis and two‐sample MR analysis. One‐sample MR analysis was carried out by using an unweighted polygenic risk score constructed by up to 27 genetic predictors as instrumental variables. Two‐sample MR analysis was carried out with up to nine genetic predictors and their effect sizes from the LifeLines‐DEEP cohort (952 samples) and the GEnetic factors for OSteoporosis (GEFOS) summary statistics (ALM sample size = 28 330). CI, confidence interval; IVW, inverse variance‐weighted. (*B*) Schematic representation of the MR analysis results: genetic predisposition to higher relative abundance of gut microbial synthesis of the SCFA butyrate (M00307: pyruvate oxidation, pyruvate → acetyl‐CoA) is associated with increased ALM.

By leveraging the summary statistic GWAS data and instruments for gut microbial synthesis of the SCFA butyrate from a recent study,^9^ we conducted two‐sample MR analysis to investigate the relationship of gut microbial synthesis of the SCFA butyrate with SMM traits. The details of summary statistic data for two‐sample MR analysis were presented in *Table* S1. The genetic variants for gut microbial synthesis of the SCFA butyrate were derived from the study conducted by Sanna *et al*.,^9^ shown in *Table* S3. Using the IVW MR method, host genetic‐driven increase in gut microbial synthesis of the SCFA butyrate was associated with ALM (*β* = 0.06, 95% CI 0 to 0.13, *P* = 0.06; *Figure* 2A). Sensitivity analyses revealed consistent results when using the weighted median method. The causal estimate of MR–Egger provided a similar point estimate but with wider 95% confidence intervals. Overall, these estimates were similar in terms of direction and magnitude, and they were unlikely to have happened by chance alone (*Figure* 2A and *Table* 4). We found no evidence of horizontal pleiotropy (*P*_MR‐PRESSOGlobal_ = 0.44 and *P*_MR–Egger Intercept_ = 0.33) and heterogeneity (*P*_Cochran's *Q*_ = 0.42). Therefore, the MR analyses largely supported the causal effect between gut microbial synthesis of the SCFA butyrate and ALM (*Figure* 2B).

**Table 4:** Two‐sample results between gut microbial synthesis of the SCFA butyrate on appendicular lean mass

MR method	N‐SNP	Appendicular lean mass		
		Estimate	95% CI	*P*
MR–Egger	9	0.08	−0.19 to 0.35	0.59
MR–Egger intercept		0.04	−0.04 to 0.12	0.33
Inverse variance weighted	9	0.06	0.00 to 0.13	0.06
Weighted median	9	0.09	0.002 to 0.18	0.04

CI, confidence interval; MR, Mendelian randomization; SCFA, short‐chain fatty acid.

N‐SNP indicates the number of SNPs used as ‘instruments’ for the exposure. Primary MR result was limited to the inverse variance‐weighted method. Other methods were considered as sensitivity analysis.

## Discussion

In this study, we investigated the potential influence of HGM on SMM. Our study was well designed for a comprehensive assessment of such influence, with multi‐omics data on whole genome‐wide sequencing of the human subjects, shotgun metagenomic sequencing of gut microbiome and human serum SCFAs, and host SMM measurements, providing an excellent unique opportunity to investigate the link between HGM and SMM, by leveraging methods of association and causality analyses.

As a prerequisite for causality, we first conducted a cross‐sectional study to investigate the potential relationship between HGM and SMM. Given the effects of diet and lifestyle factors on HGM,^30^ we included the extensive data collected through questionnaires that gathered detailed medical history as well as diet and lifestyle information as the covariates in the analysis. Considering that our study was a study with moderate sample size and the *P* values were sometimes more significant for the higher microbial taxa,^10^ thus gut microbial metabolic pathways were selected a priori for metagenomic association analysis. Our results supported that in the study sample of Chinese menopausal women, HGM plays a significant role in SMM variation, which is attributable to gut microbial synthesis of SCFA butyrate. This finding was supported by previous studies.^31^, ^32^, ^33^ For example, a recent study reported that a significant reduction of *Firmicutes* and *Roseburia* spp. was observed in faecal samples from postmenopausal women compared with faecal samples from premenopausal women.^31^ As *Firmicutes* and *Roseburia* spp. were well‐known butyrate‐producing bacteria,^26^, ^27^ these bacteria may alleviate the deficiency of endogenous oestrogen and thus contributing to lower risk of disorders such as sarcopenia in postmenopausal women with higher levels of butyrate‐producing bacteria.^31^, ^33^ However, the detailed specific mechanistic link between HGM and SMM was still largely unclear and awaits further studies.

To explore the potential causal effect of gut microbial features on SMM, we conducted complementary one‐sample and two‐sample MR analyses to assess whether HGM would play a role in SMM attributable to gut microbial synthesis of SCFA butyrate. Benefited from whole genome‐wide genotyping of the same individuals in this study, we conducted one‐sample MR analysis to explore the causal association between gut microbial synthesis of SCFA butyrate and SMM traits. In this study, we used standardized and appropriate analytical protocols to conduct the mGWAS according to the actual situation of our data. In particular: (i) because of lack of proper reference database for this study sample of Chinese menopausal women (as the existing reference database is mainly based on samples of European ancestry), gut metagenomics species construction was built *de novo* without using reference genomes as described by Nielsen *et al*.^34^; (ii) we performed a standard mGWAS with a multivariate linear or logistic model instead of the Spearman correlation method, with the covariates: age and 14 diet and lifestyle factors, as well as Top 4 PCs; and (iii) one‐sample MR analysis suggested that the increased capacity for gut microbial synthesis of SCFA butyrate was associated with ALM. On the other hand, our study showed that, even in a study of moderate sample size, the well‐designed, standardized, and appropriate analytical protocols allowed to detect human host–microbiome associations and potential causality.

The causal effect between gut microbial synthesis of the SCFA butyrate and ALM was further confirmed in independent large cohorts of European‐ancestry individuals. Leveraging GWAS summary statistics from European individuals for HGM and ALM to explore causality,^9^, ^13^ two‐sample MR results found positive relationships of gut microbial synthesis of SCFA butyrate on ALM. Although two‐sample MR analysis maximized the sample size and power to detect robust causal effect estimates, and can prevent potential confounding factors,^8^ the results should be explained with care. First, the instruments should have no horizontal pleiotropy; therefore, we applied several different MR methods. Consistency of the results across the methods that make different assumptions about pleiotropy strengthened our causal inference. Second, the selected SNPs should be suitable as the instruments. Here, the mGWAS,^9^ in which we selected the instruments, was under large‐scale design to date and thus provided powerful instrumental variables for the MR analysis.

Previous studies have clearly shown that the main butyrate‐producing bacteria are anaerobes; the low O_2_ concentrations in gut provide a favourable niche for them.^26^, ^35^ Depletion of anaerobic bacteria (induced by antibiotics, e.g.) is associated to a reduction in butyrate levels, promoting an aerobic environment and inhibiting the growth of the butyrate‐producing bacteria.^36^, ^37^ Thus, depletion of butyrate‐producing bacteria by antibiotic treatment may negatively affect SMM traits.^38^, ^39^ On the other hand, raising the amount of the butyrate‐producing bacteria could contribute to improved SMM due to the production of butyrate. As *F. prausnitzii* is one of the most common bacteria (9% of the bacteria in our study) in the gut and one of the main butyrate producers,^40^ we could stimulate the increase of the *F. prausnitzii* in individuals with low *F. prausnitzii* levels through personalized precision nutrition to improve SMM.^41^, ^42^ For example, increasing fibre intake could significantly increase the number of *F. prausnitzii*, which ferment fibres and produce butyrate.^43^

This study also had some limitations. First, our metagenomic association and one‐sample MR analyses were carried out specifically in Chinese menopausal women (average age 53 years old). Therefore, our results may not necessarily be generalized to other populations. Interestingly, two‐sample MR results partially confirmed our findings by using publicly available summary statistics data from independent larger cohorts of European individuals (both male and female), supporting that our major findings may extend to individuals of European descent. Second, the relationship of HGM on SMM detected in this study was limited; much larger population sizes would be required to comprehensively reveal more potential relationships between HGM and SMM. Third, the skeletal muscle mass and blood butyrate of host may be influenced by dietary factors, such as dietary protein level, dietary fibre, and dietary fatty acid. Unfortunately, dietary factors were not collected in the current study, and hence, we could not adjust these dietary factors as covariates in the analysis. However, the association of gut microbial synthesis of the SCFA butyrate on SMM was confirmed by MR analysis, which suggested that our findings may be robust to unassayed and uncontrolled dietary factors.

In conclusion, we confirmed the key role of gut microbial synthesis of the SCFA butyrate on host SMM by leveraging the information from 482 menopausal individuals for whom whole genome‐wide genotyping, shotgun metagenomic sequencing, and serum SCFA levels were available, also combining this information with GWAS summary statistics for HGM and ALM. Our results may help the future development of novel intervention approaches for preserving or preventing/alleviating loss of SMM.

## Author contributions

W.‐Q.L., C.‐L.G., and F.‐Y.L. contributed in the data curation; W.‐Q.L., X.L., H.‐M.L., X.Q., B.‐Y.L., W.‐D.S., C.‐L.G., and F.‐Y.L. in the formal analysis; H.‐W.D. in project conceiving, designing, and initiating; X.L. and H.‐W.D. in project administration; J.S., H.‐M.X., and H.‐W.D. in supervision; W.‐Q.L. in writing of the original draft; and W.‐Q.L., H.S., X.Q., J.S., H.‐M.X., and H.‐W.D. in writing of the review and editing.

## Conflict of interest

None declared.

## Funding

H.‐W.D. was partially supported by grants from the National Institutes of Health (U19AG05537301, R01AR069055, P20GM109036, R01MH104680, R01AG061917, and U54MD007595). J.S. was partially supported by grants from the Science and Technology Program of Guangzhou, China (201604020007), and the National Natural Science Foundation of China (81770878). H.‐M.X. was partially supported by the National Basic Research Program of China (973 Program) (2017YFC1001100 and 2016YFC1201805).

## Supporting information


**Table S1.** Genetic predictors of M00307 and their association coefficients estimated by our GWAS analysis.
**Table S2.** The characteristics of the studies for the summary‐statistic data.
**Table S3.** Genetic predictors of gut microbial synthesis of the SCFA butyrate and their association coefficients estimated by Serena Sanna et al GWAS analysis.
