# Supplementary material for: Human gut microbiome impacts skeletal muscle mass via gut microbial synthesis of the short‐chain fatty acid butyrate among healthy menopausal women
Source: J Cachexia Sarcopenia Muscle. 2021 Sep 1;12(6):1860–70. doi: 10.1002/jcsm.12788 (PMC8718076; doi:10.1002/jcsm.12788)
Supplement: Supplementary file 1 — Table S1. Genetic predictors of M00307 and their association coefficients estimated by our GWAS analysis. Table S2. The characteristics of the studies for the summary‐statistic data. Table S3. Genetic predictors of gut microbial synthesis of the SCFA butyrate and their association coefficients estimated by Serena Sanna et al GWAS analysis. [file JCSM-12-1860-s001.docx]

# Supplementary File

## Study subjects

All the individuals were recruited at the Third Affiliated Hospital of Southern Medical University, Guangzhou, Guangdong province, China. This study was approved by the Third Affiliated Hospital of Southern Medical University at Guangzhou, and all participants provided written informed consent at enrolment.

Only the menopausal women (experiencing an absence of menstruation or amenorrhea for at least 3 months; self-reported), who agreed to participate and signed the written consent form were included in the study. Those who did not provide the necessary information for the study, who received antibiotics, estrogens, or anticonvulsant drugs within 2 months before sample collection, who presented orthopedic limitations, alcoholic habits, any stage of kidney disease, and/or were receiving hormone replacement were excluded.

The individuals were recruited during a physical examination, including 518 individuals with blood samples and of which 502 had fecal samples. All these individuals were enlisted for high-depth whole genome sequencing and whole metagenomic sequencing. Blood samples were collected after an overnight fast for > 8 hours, DNA was extracted using the SolPure DNA Kit (Magen, Guangzhou, China) according to the manufacturer's protocol. Faecal samples were frozen at -80°C within 30 min of sample procurement until further analyses. Stool DNA was extracted using the E.Z.N.A.® Stool DNA Kit (Omega, Norcross, GA, USA).

## Individual-level phenotype data

With the use of specific anatomic landmarks, regions of the trunk, arms, and legs were assessed using whole-body dual-energy X-ray absorptiometry (Lunar, GE Healthcare, USA). Encore software (version 13.31.016, GE Medical Systems Lunar 3030 Ohmeda Drive Madison, WI 53718, USA) provides estimates of lean tissue mass for the total body and for standard body regions. The individuals wore light and comfortable clothes without the presence of metal objects. The equipment was used manually and all analyses were performed by the same researcher. Weight and height were measured to calculate body mass index (BMI; kg/m^2^). In our analyses, we focused on appendicular lean mass (ALM). As skeletal muscle mass is significantly correlated with body size, muscle mass is also assessed by skeletal muscle index (SMI), calculated as ALM in kilograms divided by BMI. Age, gender, diet and lifestyle factors were self-reported.

## Individual-level genotype data

In this study, 500 individuals were sequenced to a mean of 15x for whole genome sequencing. 200 ng of input DNA from blood samples were used for library preparation and then processed for paired-end 100 bp and single-end 100 bp sequencing using BGISEQ-500 platform. The reads were aligned to the latest reference human genome GRCh38/hg38 with BWA60 (version 0.7.15) with default parameters. The reads consisting of base quality < 5 or containing adaptor sequences were filtered out. The alignments were indexed in the BAM format using Samtools (version 0.1.18) and PCR duplicates were marked for downstream filtering using Picardtools (version 1.62). The Genome Analysis Toolkit's (GATK62, version 3.8) BaseRecalibrator created recalibration tables to screen known SNPs and INDELs in the BAM files from dbSNP (version 150). GATKlite (V2.2.15) was used for subsequent base quality recalibrationand removal of read pairs with improperly aligned segments as determined by Stampy. GATK's HaplotypeCaller were used for variant discovery. GVCFs containing SNVs and INDELs from GATK HaplotypeCaller were combined (CombineGVCFs), genotyped (GenotypeGVCFs), variant score recalibrated (VariantRecalibrator) and filtered (Apply Recalibration). During the GATK VariantRecalibrator process, we took our variants as inputs and used four standard SNP sets to train the model: (1) HapMap3.3 SNPs; (2) dbSNP build 150 SNPs; (3)1000 Genomes Project SNPs from Omni 2.5 chip; and (4) 1000G phase1 high confidence SNPs. The sensitivity threshold of 99.9% to SNPs and 99% to INDELs were applied for variant selection after optimizing for Transition to Transversion (TiTv) ratios using the GATK ApplyRecalibration command. There were 3,2505,582 raw variants left after applying the recalibration.

We applied a conservative inclusion threshold for variants: (i) Hardy-Weinberg equilibrium (HWE) P > 1ⅹ10^-5^; and (ii) genotype calling rate > 95%. We demanded samples to meet these criteria: (i) variant calling rate > 95%; (ii) no population stratification by performing principal components analysis (PCA) analysis implemented in PLINK (version 1.9) and (iii) excluding related individuals by calculating pairwise identity by descent (IBD, Pi-hat threshold of 0.2) in PLINK. After variant and sample quality control 482 individuals with 7.39 million common (MAF ≥ 1%) variants were left for subsequent analyses.

## Individual-level metagenomic sequencing and profiling data

High-quality whole metagenomic sequencing was performed for 502 samples with fecal samples available. Construction of a paired-end (PE) library with insert size of 300 bp was performed according to the manufacturer’s instruction, and the DNA library was sequenced with PE reads of 2×150 bp on the Illumina Hiseq4000 platform. Raw sequencing reads were processed to obtain valid reads for further analysis. First, sequencing adapters were removed from sequencing reads using cutadapt (version 1.9). Secondly, low quality reads were trimmed by fqtrim (version 0.94) using a sliding-window algorithm. Thirdly, reads were aligned to the host genome using bowtie2 (version 2.2.0) to remove host contamination. About library-size correction, we downsized the unique reads to 40 million for each sample to eliminate the influence of sequencing depth in comparative analyses. Once quality-filtered reads were obtained, they were de novo assembled to construct the metagenome for each sample by SPAdes (version 3.10.0). All coding regions (CDS) of metagenomic contigs were predicted by MetaGeneMark (version 3.26). CDS sequences of all samples were clustered by CD-HIT (version 4.6.1) to obtain unigenes.

After quality control and host gene removal, clean reads were mapped to the 7,792,376 unigenes in the integrated gene catalogue with a threshold of more than 90% identity over 95% of the length. Unigenes abundance for a certain sample were estimated by transcripts per kilobase million (TPM) based on the number of aligned reads by bowtie2 (version 2.2.0). KEGG Orthology (KO) gene group abundance profiles were calculated by summing the abundance of genes annotated to the respective KO gene group. KOs were mapped to microbiome functional KEGG modules based on annotations from the KEGG BRITE database. The unigenes were clustered by co-abundance as described by Nielsen *et al* (1), which defined co-abundance gene groups (CAGs) with high correlations (Pearson correlation coefficient > 0.9). The CAGs, with more than 700 genes, were considered as metagenomics species (MGS). The abundance profiles of the MGS were determined as the medium gene abundance throughout the samples. Furthermore, the MGSs were taxonomically annotated, by summing up the taxonomical annotation of their genes as described by Nielsen *et al* (1). In brief, each gene was annotated by sequence similarity to known reference genomes, sequence similarity of 95%, 85% and 75% or better was used for species, genus and phylum level taxonomical annotation, respectively. MGS were assigned a species level annotation if more than 50% of the genes comprising the CAG were assigned a given species level taxonomy (including genes with no match). MGS were described to have 'clear and unambiguous similarity to a known species' when 90% or more of the genes were annotated to the same species. The CAGs that could not be assigned to a genus or species by DNA similarity were in addition taxonomically annotated by similarity to the UniProt database (BLASTP, best hit, E < 0.001) to get an approximate taxonomical annotation. Gut metabolic modules reflect bacterial and archaeal metabolism specific to the human gut, with a focus on anaerobic fermentation processes. The set of gut metabolic modules was built through KEGG modules, followed by expert curation and delineation of modules and alternative pathways. Finally, we identified 646 common microbial KEGG modules present in 50% or more of the samples.

## Individual-level fasting serum short chain fatty acids (SCFAs)

Serum samples were vortex-mixed with 36% phosphoric acid solution and further extracted supernatants by liquid-liquid extraction with methyl tert-butyl ether which containing SCFA stock solutions. Detailed conditions for Gas chromatography-tandem mass spectrometry (GC-MS/MS) were as follows: setting the injector temperature at 240 C, keeping the initial oven temperature at 90℃ for l min, then gradually raising it to 140℃, 160℃, 200℃, and 240℃ at a rate of 10℃/min, 5 ℃/min, 15℃/min, and 10℃/min, respectively. Pure helium was used as a carrier gas at 1.0 ml/min rate. The main conditions of mass spectrometry included electron impact ion source and multi-reaction monitoring scan mode. The temperature of the transfer line, ion source, and quad were 240℃, 230℃, and 150℃, respectively. The electron energy was 70 eV, and the solvent delayed 2.4 min.

Process of quality control (QC) and intra-day/inter-day accuracy were as follows: the QC samples were composed of SCFA stock solutions dissolved in methyl tert-butyl ether. Then the QC samples were processed in parallel with test samples to analyze detection stability and repeatability under the same process with an injection volume of 2 ul. Three QC samples were continuously injected to test the instrument stability. For every 10 test samples injected, a QC sample was inserted to check for the repeatability of the instrument. Intra-day and inter-day accuracy were evaluated and reported as CV% of repeatability at the concentration of each SCFA. The intra-day accuracy is 0.42%-3.64%, and the inter-day accuracy is 1.12%-3.40%, indicating a good stability of the instrument.

We performed qualitative and quantitative analysis of SCFAs with Agilent Mass Hunter software. By using a stock solution containing mixtures of SCFAs, five calibration standards (concentration range from 0.005 to 8 mg/L) were prepared. Then we performed GC-MS/MS measurement as described above and integrated the obtained signals (e.g, retention time and peak area) to calculate relative retention time and area ratios. Meanwhile, the calibration curves were constructed by plotting the peak area versus concentration for each individual SCFA. And the slopes of the calibration curves were determined by performing linear regression analysis. In addition, average area ratio of blank samples was used as background signal/intercept. Finally, we calculated the concentrations of SCFAs with the area ratio, average area ratio blank samples, and slope (see following formula).

$$Concentration=\frac{area ratio-average ratio blank sample}{slope}$$

# Reference

1. Nielsen H B, Almeida M, Juncker A S, Rasmussen S, Li J, Sunagawa S *et al.* Identification and assembly of genomes and genetic elements in complex metagenomic samples without using reference genomes. *Nat Biotechnol* 2014;**32**:822-8.

| Supplementary Table 1. Genetic predictors of M00307 and their association coefficients estimated by our GWAS analysis | | | | | | | | | |
| --- | --- | --- | --- | --- | --- | --- | --- | --- | --- |
| SNP | CHR | BP | A1 | A2 | EAF | beta | SE | *P* | F |
| **rs151213951** | **14** | **18681491** | **G** | **T** | **0.012** | **0.381** | **0.067** | **2.27E-08** | **32.3** |
| rs1020822 | 3 | 53715707 | G | C | 0.890 | -0.102 | 0.022 | 5.84E-06 | 21.0 |
| rs10273830 | 7 | 26433169 | T | C | 0.646 | -0.071 | 0.016 | 7.65E-06 | 20.4 |
| rs10894565 | 11 | 132484500 | G | A | 0.805 | -0.088 | 0.018 | 2.36E-06 | 22.8 |
| rs10928240 | 2 | 145062903 | C | G | 0.761 | -0.084 | 0.017 | 1.53E-06 | 23.6 |
| rs10977440 | 9 | 9007899 | T | C | 0.459 | 0.066 | 0.014 | 6.11E-06 | 20.9 |
| rs112249721 | 10 | 45001188 | A | C | 0.139 | -0.094 | 0.021 | 6.72E-06 | 20.7 |
| rs117012479 | 15 | 69056874 | G | C | 0.028 | 0.195 | 0.043 | 6.16E-06 | 20.8 |
| rs117184532 | 22 | 26302861 | T | C | 0.021 | 0.241 | 0.051 | 3.72E-06 | 21.9 |
| rs11756879 | 6 | 166090298 | A | T | 0.986 | -0.274 | 0.059 | 4.05E-06 | 21.7 |
| rs12040185 | 1 | 247450200 | A | C | 0.277 | 0.073 | 0.016 | 6.39E-06 | 20.8 |
| rs146108854 | 19 | 19150346 | A | G | 0.067 | 0.152 | 0.029 | 2.30E-07 | 27.5 |
| rs1503841 | 3 | 100839156 | G | A | 0.495 | 0.067 | 0.015 | 6.47E-06 | 20.7 |
| rs182794294 | 3 | 113573302 | C | T | 0.016 | 0.274 | 0.058 | 3.45E-06 | 22.0 |
| rs28969454 | 12 | 1626627 | T | C | 0.897 | -0.110 | 0.023 | 1.71E-06 | 23.4 |
| rs35447764 | 2 | 174506489 | C | T | 0.013 | 0.303 | 0.066 | 5.33E-06 | 21.1 |
| rs371300737 | 2 | 87347502 | G | A | 0.013 | 0.306 | 0.066 | 4.58E-06 | 21.4 |
| rs4614427 | 11 | 13398472 | G | C | 0.231 | 0.084 | 0.018 | 2.62E-06 | 22.6 |
| rs4708909 | 6 | 161413957 | C | G | 0.858 | -0.101 | 0.022 | 4.11E-06 | 21.7 |
| rs60907448 | 4 | 164559358 | T | C | 0.281 | 0.078 | 0.016 | 2.26E-06 | 22.9 |
| rs62537076 | 9 | 40140751 | T | C | 0.066 | 0.138 | 0.029 | 3.81E-06 | 21.8 |
| rs6456551 | 6 | 23446719 | T | C | 0.011 | 0.334 | 0.072 | 4.49E-06 | 21.5 |
| rs74544987 | 10 | 99792678 | A | T | 0.019 | 0.249 | 0.054 | 5.03E-06 | 21.2 |
| rs75541286 | 7 | 2766997 | C | A | 0.023 | 0.230 | 0.049 | 3.75E-06 | 21.8 |
| rs79577416 | 13 | 76320013 | T | C | 0.975 | -0.228 | 0.050 | 6.35E-06 | 20.8 |
| rs80056132 | 18 | 57161479 | C | T | 0.041 | 0.173 | 0.037 | 4.91E-06 | 21.3 |
| rs9465301 | 6 | 19224074 | G | A | 0.133 | 0.108 | 0.022 | 8.36E-07 | 24.9 |

SNP, single-nucleotide polymorphisms; CHR, chromosome; BP, base position; A1, effect allele; A2, non-effect allele; EAF = effect allele frequency; beta, coefficient for A1; SE, standard error.

| Supplementary Table 2. The characteristics of the studies for the summary-statistic data | | | | | | | |  |
| --- | --- | --- | --- | --- | --- | --- | --- | --- |
| **Cohort** | **Phenotype** | **Ethnicities** | | **Sample size** | | **Web source** | |  |
| LifeLines-DEEP (LL-DEEP) cohort | Gut microbial synthesis of SCFA butyrate | European | | 952 | | <https://www.nature.com/articles/s41588-019-0350-x#Sec3> | |  |
|  |  |  | |  | |  | |  |
| GEnetic Factors for OSteoporosis Consortium (GEFOS) | Appendicular lean mass | | European | | 28,330 | | http://www.gefos.org/?q=content/adult-lean-mass-gwas-2017 | |

| Supplementary Table 3. Genetic predictors of gut microbial synthesis of the SCFA butyrate and their association coefficients estimated by Serena Sanna *et al* GWAS analysis | | | | | | |
| --- | --- | --- | --- | --- | --- | --- |
| SNP | A1 | A2 | EAF | beta | SE | *P* |
| rs9423658 | C | T | 0.86 | 0.33 | 0.071 | 5.7E-06 |
| rs881390 | C | T | 0.11 | 0.4 | 0.087 | 5.7E-06 |
| rs2089222 | A | G | 0.04 | 0.56 | 0.122 | 3.7E-06 |
| rs9904981 | G | A | 0.77 | 0.25 | 0.056 | 6.0E-06 |
| rs10483112 | T | C | 0.03 | 0.59 | 0.128 | 5.2E-06 |
| rs12994030 | T | C | 0.27 | 0.24 | 0.051 | 3.5E-06 |
| rs2056208 | T | C | 0.24 | 0.24 | 0.051 | 9.1E-06 |
| rs10019739 | C | T | 0.27 | 0.24 | 0.056 | 8.2E-06 |
| rs7743827 | G | A | 0.80 | 0.27 | 0.061 | 4.9E-06 |

SNP, single-nucleotide polymorphisms; A1, effect allele; A2, non-effect allele; EAF = effect allele frequency; beta, coefficient for A1; SE, standard error.

# Reference

Sanna S, van Zuydam N R, Mahajan A, Kurilshikov A, Vich Vila A, Vosa U *et al.* Causal relationships among the gut microbiome, short-chain fatty acids and metabolic diseases. *Nat Genet* 2019;**51**:600-05.
